# Safety of Thioguanine in Pediatric Inflammatory Bowel Disease: A Multi-Center Case Series

**DOI:** 10.1097/MPG.0000000000003621

**Published:** 2022-09-20

**Authors:** Ahmed B. Bayoumy, Jasmijn Z. Jagt, Herbert M. van Wering, Lissy de Ridder, Thalia Hummel, Victorien M. Wolters, Janneke Stapelbroek, Marc A. Benninga, Chris J.J. Mulder, Nanne K.H. de Boer, Tim G.J. de Meij

**Affiliations:** From the * Faculty of Medicine, Amsterdam UMC, University of Amsterdam, Amsterdam, the Netherlands; the † Department of Pediatric Gastroenterology, Emma Children’s Hospital, Amsterdam UMC, Vrije Universiteit Amsterdam, Amsterdam, the Netherlands; the ‡ Amsterdam UMC, Vrije Universiteit Amsterdam, Paediatric Gastroenterology, Amsterdam Gastroenterology Endocrinology Metabolism, Amsterdam, the Netherlands; the § Department of Pediatric Gastroenterology, Amphia Hospital, Breda, the Netherlands; the ‖ Department of Pediatric Gastroenterology, Erasmus MC Sophia Children’s Hospital, Rotterdam, the Netherlands; the ¶ Department of Pediatric Gastroenterology, Medisch Spectrum Twente, Enschede, the Netherlands; the # Department of Pediatric Gastroenterology, Wilhelmina Children’s Hospital, University Medical Center Utrecht, Utrecht, the Netherlands; the ** Department of Pediatric Gastroenterology, Catharina Hospital, Eindhoven, the Netherlands; the †† Department of Pediatric Gastroenterology and Nutrition, Emma Children’s Hospital, Amsterdam University Medical Centers, University of Amsterdam, Amsterdam, the Netherlands; the ‡‡ Department of Gastroenterology and Hepatology, Amsterdam Gastroenterology and Metabolism Research Institute, Amsterdam UMC, Vrije Universiteit Amsterdam, Amsterdam, the Netherlands.

**Keywords:** inflammatory bowel disease, pediatrics, thioguanine, thiopurines

## Abstract

Thioguanine (TG) has been shown as a safe alternative in adults with inflammatory bowel disease (IBD) who did not tolerate conventional thiopurines [azathioprine (AZA)/mercaptopurine]. However, data in pediatric IBD are scarce. Therefore, we aimed to assess the safety of TG as maintenance therapy.

**Methods:**

A retrospective, multicenter cohort study of children with IBD on TG was performed in the Netherlands. TG-related adverse events (AE) were assessed and listed according to the common terminology criteria for AE.

**Results:**

Thirty-six children with IBD (median age 14.5 years) on TG (median dose 15 mg/day) were included in 6 centers. Five AE occurred during follow-up [pancreatitis (grade 3), hepatotoxicity (grade 3) (n = 2), *Clostridium difficile* infection (grade 2), and abdominal pain (grade 2)]. All patients (n = 8) with a previously AZA-induced pancreatitis did not redevelop pancreatitis on TG.

**Conclusions:**

In pediatric IBD, TG seems a safe alternative in case of AZA-induced pancreatitis. Further research assessing long-term TG-related safety and efficacy is needed.

**What Is Known**

- Thioguanine (TG) has been shown as a safe and effective alternative thiopurine in adults with inflammatory bowel disease (IBD).
- Data on the safety profile of TG in pediatric IBD are limited.

**What Is New**

- No pancreatitis occurred in all 8 patients with a previously azathioprine (AZA)-induced pancreatitis.
- Our results suggest that TG might especially serve as a proper alternative in pediatric IBD patients with previously AZA-induced pancreatitis.

Conventional drug therapies (such as thiopurines and methotrexate) are used in pediatric inflammatory bowel disease (IBD), both as primary maintenance therapy and combined with anti-tumor necrosis factor (TNF) treatment treatment to reduce the risk of developing anti-drug antibodies. However, a subset of children do not respond to or tolerate these agents (1,2). In addition, concerns exist on a possible higher risk of malignancy in patients exposed to azathioprine (AZA) and mercaptopurine (MP), which has resulted in a decreased use of these conventional thiopurines (3). Hence, there is a need for safe alternative maintenance treatment options. Since 2022, thioguanine (TG) has been formally licensed in the Netherlands for the treatment of adult IBD patients (4,5). The metabolic advantage of TG compared to AZA and MP is that it is directly converted to biologically active 6-thioguanine nucleotides (6-TGN) (6), without producing the potentially toxic metabolite 6-methylmercaptopurine (6-MMP). Nonetheless, TG use is limited especially in children due to earlier concerns on liver toxicity, specifically non-cirrhotic portal hypertension (NCPH) (7). Ongoing reports observed that the incidence of nodular regenerative hyperplasia (NRH) of the liver was approximately 2%–6% in proper-dosed TG-treated adults, which is similar to the background incidence of NRH in the adult IBD population (8). In adult IBD, proper-dosed TG (0.2–0.3 mg/kg/day) has been considered an effective and safe alternative as rescue therapy (9). However, data on TG in pediatric IBD are scarce. The aim of this study was to assess the safety of TG as rescue maintenance therapy in children with IBD.

## METHODS

### Study Design and Patient Population

A retrospective, multicenter case series was performed on behalf of the Kids Crohn’s and Colitis (KiCC) working group for Collaborative Research in the Netherlands. All KiCC members, working in 15 different centers across the Netherlands, were asked to identify children with IBD (previously) using TG. Patients were eligible to participate if they were <18 years old at initiation of TG, diagnosed with Crohn disease (CD), ulcerative colitis (UC), or IBD-unclassified (IBD-U) according to the revised Porto criteria (10). Patients were excluded if no follow-up was available.

### Data Collection

Patient characteristics, pharmacological, and surgical history were collected. In addition, biochemical, radiological, and histopathological data were collected within the interval of initiation of TG until final follow-up. Laboratory data were collected at assessment of 6-TGN levels, including hemoglobin, mean corpuscular volume, leukocytes, thrombocytes, aspartate aminotransferase (ASAT), alanine aminotransferase (ALAT), erythrocyte sedimentation rate, C-reactive protein, alkaline phosphatase (AP), gamma glutamyl-transferase (gamma GT), albumin, and fecal calprotectin. The concentrations of 6-TGN in all participating centers were determined in red blood cells, using a method (modified) from Dervieux and Boulieu (11). Thiopurine methyltransferase (TPMT) status, if available, was collected. The use of biological agents was documented.

### Safety Assessment

The occurrence of adverse events (AE) and reasons for withdrawal during TG treatment were recorded during the entire duration of follow-up. The end of follow-up was defined as discontinuation of TG or last clinical visit on TG. AE were defined as laboratory abnormalities and signs or symptoms that occurred after initiation of TG, and were listed according to the common terminology criteria for AE (version 5.0, released November 27, 2017) (12). Leukopenia was defined as leukocytes below 3.0 × 10^9^/L. Liver toxicity was evaluated by radiological and biochemical parameters. Hepatotoxicity was defined as twice upper limit of ALAT, ASAT, AP, or gamma GT. Furthermore, emphasis has been put on clinical signs or imaging findings of NCPH (eg, hepato- or splenomegaly, ascites, nodular abnormalities, varices) in medical records. Correlations between 6-TGN levels [pmol/8 × 10^8^ red blood cells (RBC)] and laboratory parameters including hematological indices and liver tests were assessed.

### Statistical Analysis

Data were presented as numbers with percentages, medians with interquartile range (IQR), or means with standard deviations. Depending on the kind of parameter and distribution, parametric or nonparametric tests including the Mann-Whitney *U* test, Wilcoxon signed rank test, Kruskal Wallis, and the Student *t*-test or chi-square test were used to test for differences within and between groups. This study was reported according to the Strengthening the Reporting of Observational Studies in Epidemiology statement (13). IBM SPSS Statistics for Windows, Version 26.0 (IBM Corp, Armonk, NY, USA) was used for the statistical analysis. A *P* value less than 0.05 was considered as statistically significant.

### Ethical Consideration

The study protocol was approved on October 2020 by the Medical Ethical Review Committee of VU University Medical Center under file number 2020.401, and by the ethical committees of all participating centers. All study participants gave written informed consent before data were collected.

## RESULTS

### Patients’ Characteristics

Thirty-six patients with IBD (24 CD, 9 UC, and 3 IBD-U, 56% female) on TG were included in 6 centers in the Netherlands (Amsterdam UMC, Erasmus MC, UMC Utrecht, Amphia Hospital, Catharina Hospital Eindhoven, and Medisch Spectrum Twente). All patients started with TG [median dosage 15 mg/day (IQR 10–20)] after AZA failure (83% side-effects, 6% nonresponse, and 11% high 6-MMP/low 6-TGN levels). The median age of patients at start of TG was 14.5 (IQR 11.3–16.0) years. The median duration of follow-up was 49 (IQR 23–77) weeks. One patient suffered from extra-intestinal manifestations (auto-immune hepatitis). The summary of patients’ characteristics is depicted in Table 1. Of the 36 patients on TG, 10 patients were on TG monotherapy (28%) and 26 patients were exposed to biologicals (72%) (Figure 1, Supplemental Digital Content, http://links.lww.com/MPG/C943). Eleven (31%) patients stopped TG treatment during follow-up due to primary nonresponse (n = 4), side-effects (n = 4), and TG withdrawal following a period of combination therapy with infliximab (n = 3).

**TABLE 1.** Baseline characteristics of included patients

Parameter	Total cohort (n = 36)
Age at start of TG (median, IQR)	14.5 yr (11.3–16.0)
Sex (male:female)	16:20
IBD type (CD:UC:IBD-U)	24:9:3
Classification	
CD	
Age (A1a:A1b:A2)	3:20:1
Location (L1:L2:L3:L4a:L4b)	7:4:9:11:2
Behavior (B1:B2:B3:B2B3)	21:1:1:1
Perianal disease	3 (13%)
Growth delay	4 (18%)
UC	
Extent (E1: E2: E3: E4)	0: 1: 0: 7
Severity (S0: S1)	1: 8
Extra-intestinal manifestations (yes, %)	1 (3)
Surgery pre-TG (yes, %)	2 (6)
Short bowel disease (yes, %)	0 (0)
TG dosage (median, IQR)	15 mg/day (IQR 10–20)
TPMT measured (%)	21 (57)
*1/*1 (wild-type, normal metabolism)	10 (47)
*1/*2 (variant, intermediate metabolism)	11 (53)
Prior treatments	
Nutritional therapies	18 (50%)
EEN	15 (42%)
SCD	1 (3%)
CDED	2 (6%)
Medical therapies	
Azathioprine	36 (100%)
Mercaptopurine	3 (8%)
Corticosteroids	16 (44%)
Allopurinol-thiopurine combination	4 (11%)
Infliximab	14 (39%)
Methotrexate	1 (3%)
Mesalazine	11 (31%)
Duration of follow-up	49 wk (IQR 23–77)
Duration of TG use (median, IQR)	48 wk (IQR 23–75)

CD = Crohn disease; CDED = Crohn disease exclusion diet; EEN = exclusive enteral nutrition; IBD-U = IBD-unclassified; IQR = interquartile range; SCD = specific carbohydrate diet; TG = thioguanine; TPMT = thiopurine methyltransferase; UC = ulcerative colitis.

### Safety

Five AE were reported in 4 of 36 patients (11%), including pancreatitis (grade 3), hepatotoxicity (grade 3) (n = 2), *Clostridium difficile* infection (grade 2), and abdominal pain (grade 2). TG was stopped in the patient with pancreatitis at 23 weeks following initiation of TG, in the patient with abdominal pain at 8 weeks, and in the 2 patients with hepatotoxicity (13 and 15 weeks after start of TG). No life-threatening AE, malignancies, and leukopenia occurred during follow-up. No liver biopsies were performed during TG use. No clinical signs of esophageal varices (eg, hematemesis or melena) and hepatosplenomegaly—related to NCPH—were reported. Abdominal ultrasound was performed in 8 patients (22%) and no abnormalities were reported. Table 2 shows the outcomes of TG use after cessation of AZA therapy. All patients (n = 8) who suffered from AZA-induced pancreatitis did not redevelop pancreatitis while using TG.

**TABLE 2.** Outcome of thioguanine use after cessation of azathioprine therapy in this cohort (n = 36)

Drug	Stop reason	Specified	Drug	Outcome of TG	Specified
AZA	Side-effect	Pancreatitis	TG	Continued use	
AZA	Side-effect	Pancreatitis	TG	Continued use	
AZA	Side-effect	Pancreatitis	TG	Continued use	
AZA	Side-effect	Pancreatitis	TG	Continued use	
AZA	Side-effect	Pancreatitis	TG	Nonresponse	
AZA	Side-effect	Pancreatitis	TG	Side-effect	Abdominal pain
AZA	Side-effect	Pancreatitis	TG	Remission	
AZA	Side-effect	Pancreatitis	TG	Remission	
AZA	Side-effect	Lipase↑	TG	Side-effect	Pancreatitis
AZA	Side-effect	Lipase↑	TG	Nonresponse	Severe colitis
AZA	Side-effect	LFT↑	TG	Continued use	
AZA	Side-effect	LFT↑	TG	Remission	
AZA	Side-effect	LFT↑	TG	Side-effect	LFT↑
AZA	Side-effect	Nausea	TG	Nonresponse	
AZA	Side-effect	Nausea	TG	Continued use	
AZA	Side-effect	Nausea	TG	Continued use	
AZA	Side-effect	Nausea	TG	Continued use	
AZA	Side-effect	Nausea	TG	Continued use	
AZA	Side-effect	Hypotension and elevated amylase	TG	Continued use	
AZA	6-TGN	High 6-MMP, low 6-TGN	TG	Continued use	
AZA	6-TGN	High 6-MMP, low 6-TGN	TG	Continued use	
AZA	6-TGN	Low 6-TGN	TG	Side-effect	LFT↑
AZA	6-TGN	Low 6-TGN	TG	Continued use	
AZA	Side-effect	Headache	TG	Continued use	
AZA	Side-effect	Hair loss	TG	Continued use	
AZA	Side-effect	Extreme fatigue	TG	Continued use	
AZA	Side-effect	Arthralgia	TG	Continued use	
AZA	Side-effect	Arthralgia	TG	Continued use	
AZA	Side-effect	Arthralgia	TG	Continued use	
AZA	Side-effect	No specified	TG	Continued use	
AZA	Side-effect	No specified	TG	Continued use	
AZA	Side-effect	No specified	TG	Continued use	
AZA	Side-effect	No specified	TG	Continued use	
AZA	Side-effect	No specified	TG	Continued use	
AZA	Nonresponse		TG	Nonresponse	Flare-up
AZA	Nonresponse		TG	Continued use	

AZA = azathioprine; LFT = liver function tests; MMP = methylmercaptopurine; TG = thioguanine; TGN = thioguanine nucleotides.

### Therapeutic Drug Monitoring

When a 6-TGN cut-off level was used of 1000 pmol/8 × 10^8^ RBC, significant differences were observed in ALAT, ASAT, GGT, and albumin levels between patients with 6-TGN levels ≤1000 pmol/8 × 10^8^ RBC versus patients with 6-TGN levels >1000 pmol/8 × 10^8^ RBC. The summary of results of laboratory parameters analyzed by 6-TGN cut-off level can be found in Figures 2–4, Supplemental Digital Content, http://links.lww.com/MPG/C943. All abnormal liver function tests occurred at 6-TGN levels above 1300 pmol/8 × 10^8^ RBC (Figure 5, Supplemental Digital Content, http://links.lww.com/MPG/C943). Furthermore, TPMT genotyping was performed in 21 patients, of whom 10 patients had a normal metabolism (*1/*1) and 11 patients had an intermediate metabolism (*1/*2). There was a significant difference in 6-TGN level between patients with TPMT genotype *1/*2 compared to patients with TPMT genotype *1/*1 (1083.0 vs 625.3 pmol/8 × 10^8^ RBC, *P* = 0.001) (Figure 6, Supplemental Digital Content, http://links.lww.com/MPG/C943). There was no correlation between the TPMT genotypes and the levels of ALAT, ASAT, GGT, and albumin.

## DISCUSSION

In this retrospective cohort of children with IBD on TG, side-effects were seen in 11% and no new safety signals were observed during short-term follow-up. Additionally, no pancreatitis occurred in all 8 patients with a previously AZA-induced pancreatitis.

### Safety

Concerns have been raised about the safety profile of TG in the past, especially regarding hepato- and myelotoxicity. Dubinsky et al (7) raised concerns of relatively high incidence of NRH in IBD patients using TG dosages up to 100 mg daily. In the present study comprising patients using lower TG doses (median dose 15 mg/day), no clinical features (eg, variceal bleeding, hepatosplenomegaly) related to NCPH—often caused by NRH—were observed. However, ultrasounds were performed infrequently and no liver biopsies were taken, thus no conclusions could be drawn on the incidence of histological manifestations of NRH.

Two patients in this study experienced hepatotoxicity (6%) while none developed leukopenia. This is lower as compared to the retrospective study of Dubinsky et al (7) in which 26% of children and adults with IBD experienced abnormal liver chemistry values and/or myelotoxicity. This difference could be explained by the different TG dosages used [higher dosages (≥45 mg/day) in the cohort of Dubinsky], since myelosuppression is considered a dose-dependent AE (14). However, it must be noted that the duration of follow-up in our study was limited (median 49 weeks), which may not allow to fully assess the incidence of these AE in children on low-dose TG (15). No malignancies were observed in the present study, yet due to the small patient cohort and limited duration of follow-up this study does not allow to make firm statements about the malignancy risk of TG.

Eight patients started TG after an AZA-induced pancreatitis. Interestingly, none of these patients developed pancreatitis on TG. In adults with IBD who experienced an AZA/MP-induced pancreatitis, TG is specifically considered as a proper alternative (16). However, data in children with IBD are limited so far. Our results indicate that TG should be considered as an alternative thiopurine in pediatric patients who suffered from an AZA-induced pancreatitis. In terms of short-term safety, 4 patients (11%) ceased TG due to AE (pancreatitis, hepatotoxicity, and abdominal pain). This is lower as compared to AE rates related to conventional thiopurines in pediatric IBD (17,18). A previous study in pediatric IBD patients (15) described that around 11% of patients ceased conventional thiopurines within only 2 months due to early intolerance, with gastro-intestinal and flu-like symptoms as most reported AE. This was not observed in our cohort of patients using TG therapy, which might be explained by bypassing several metabolic steps (reducing the formation of potentially toxic metabolites) (5,6).

Interestingly, 11 of 36 patients had intermediate metabolism (*1/*2), which is higher as compared to the general pediatric IBD population (19). Likely, patients who experienced side-effects on AZA had higher TGN levels because of intermediate metabolism. That would clarify the higher percentage of intermediate metabolism (*1/*2) in this cohort of pediatric IBD patients who failed AZA therapy. Also, it was found that ALAT, ASAT, and GGT levels correlated with 6-TGN levels, and that levels of these parameters were significantly higher in patients who had 6-TGN levels above 1000 pmol/8 × 10^8^ RBC. There is scarce evidence for the role of 6-TGN measurements in TG-treated IBD patients. It has been thought that the 6-TGN level should be kept below 1000 pmol/8 × 10^8^ RBC due to occurrence of NRH, which is hypothesized to occur at high 6-TGN levels. Based on the results of this study (hepatotoxicity in patients with 6-TGN levels > 1000 pmol/8 × 10^8^ RBC), 6-TGN levels could be aimed below 1000 pmol/8 × 10^8^ RBC, as is recommended for adult IBD patients (20). However, this needs further evaluation before it could be implemented in recommendations for therapeutic drug monitoring (TDM) of TG in pediatric IBD.

### Strengths and Limitations

One of the strengths of this study was the investigation of the safety of TG in pediatric IBD patients who did not tolerate or respond to conventional therapies. Furthermore, TDM data were available in the majority of patients (87%) which provides unique data on the role of 6-TGN in TG-treated pediatric IBD patients. However, this study also has some limitations that should be noted. First of all, the retrospective character of this study prevented a standardized collection of data. Another limitation of this study was the small size of the study population. Unfortunately, TG is used as not reimbursed off-label treatment in pediatric IBD patients, largely due to the lack of experience with TG in clinical practice. Therefore the number of children that are prescribed TG for IBD in the Netherlands remains limited. Furthermore, endoscopic data during TG use were scarce in this study. The last limitation was the limited duration of follow-up. Important long-term outcomes such as veno-occlusive disease, NRH and NCPH, and developmental outcome (eg, growth) could therefore not be analyzed. Hence, prospective registry studies with a long-term follow-up and standardized data collection on safety (and efficacy) are needed to assess the long-term safety profile of TG in children with IBD. This is important as TG can be used as a long-term maintenance treatment.

## CONCLUSIONS

In conclusion, our data suggest that in children with IBD, TG might serve as a proper alternative and not only in patients with previously AZA-induced pancreatitis. This study design did not allow drawing conclusions on the long-term safety profile of TG in children with IBD. Therefore, future research assessing long-term TG-related complications—especially NRH/NCPH—and on efficacy is needed.

## Supplementary Material


