# Supplementary figures and images for: Safety of Thioguanine in Pediatric Inflammatory Bowel Disease: A Multi-Center Case Series
Source: J Pediatr Gastroenterol Nutr. 2022 Sep 20;75(6):e111–5. doi: 10.1097/MPG.0000000000003621 (PMC9645549; doi:10.1097/MPG.0000000000003621)

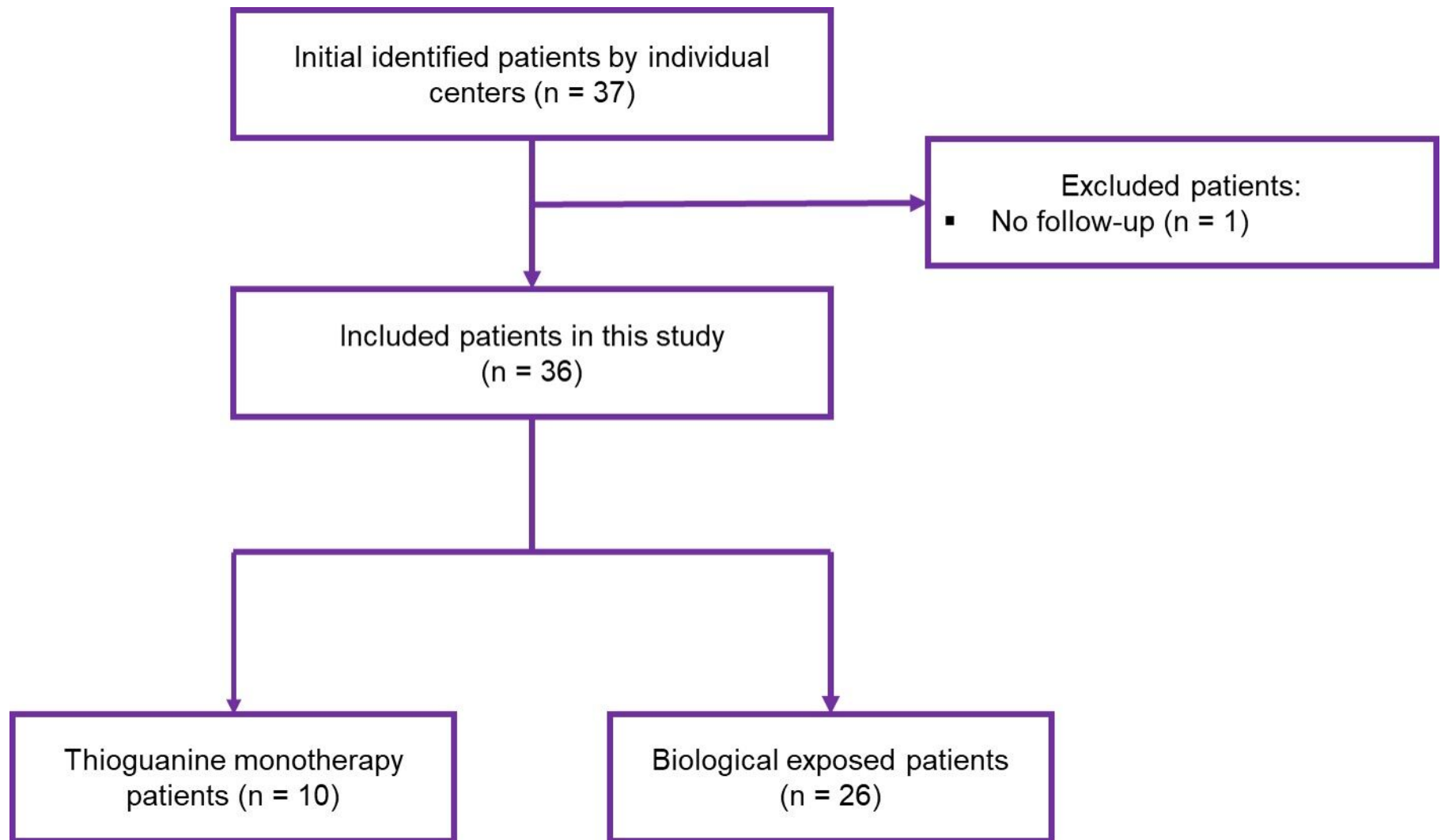

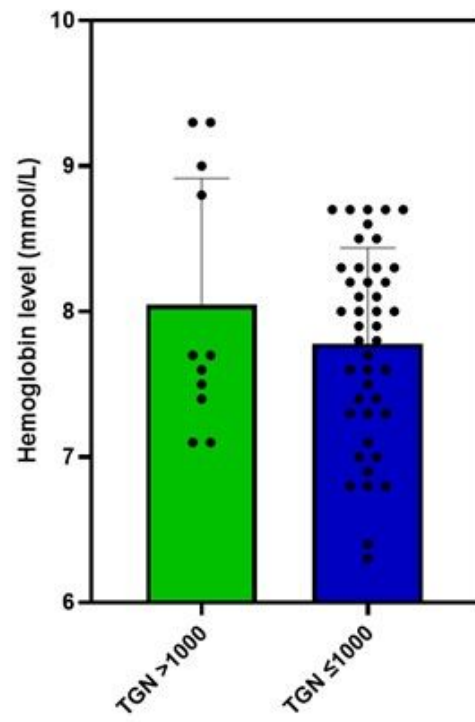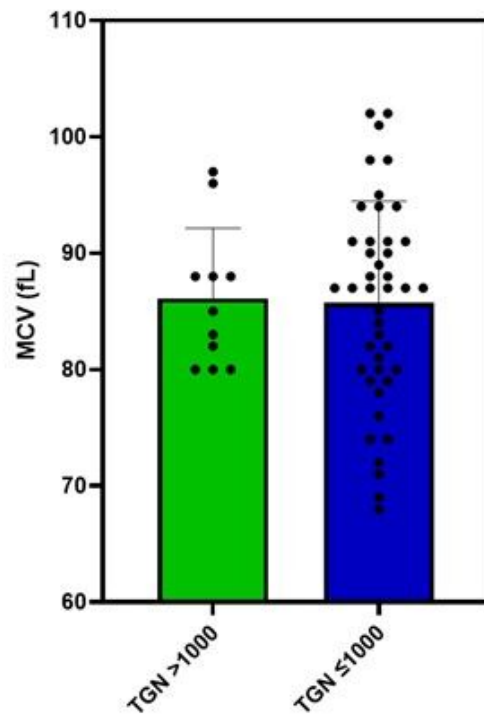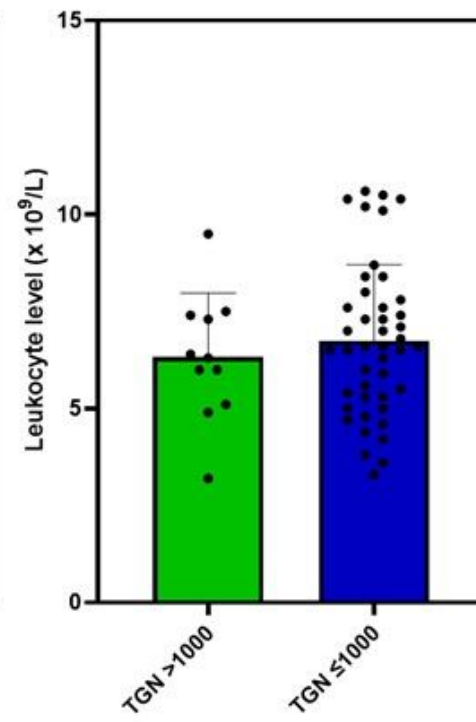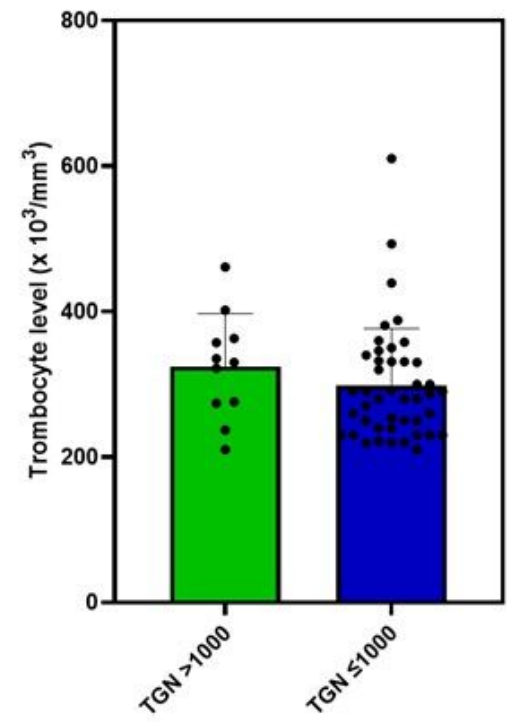

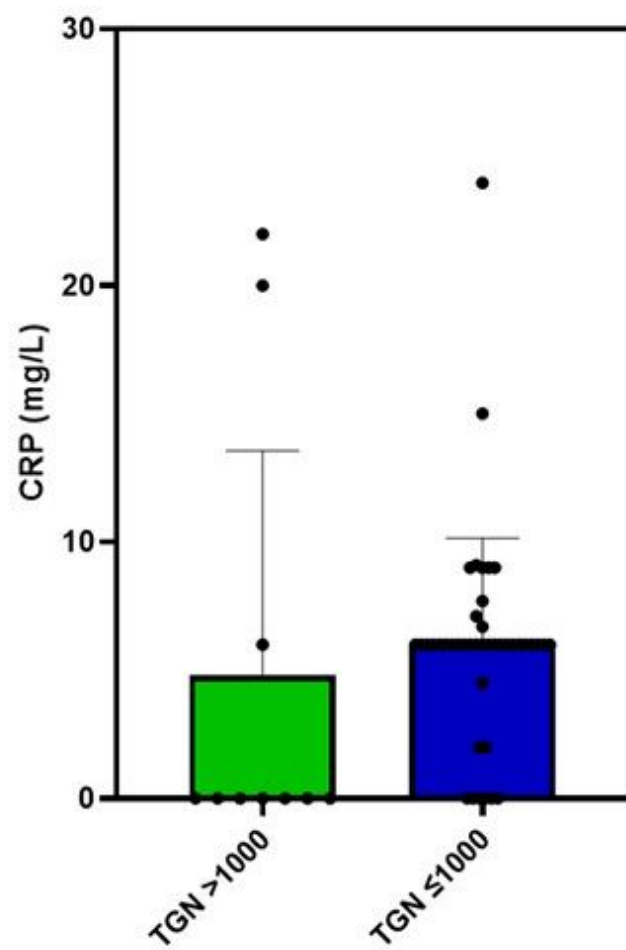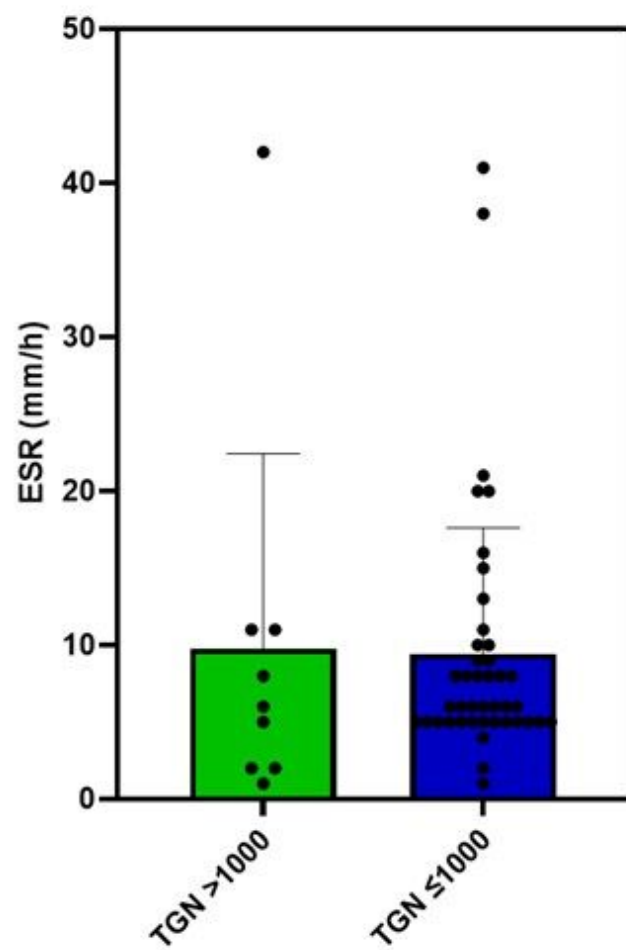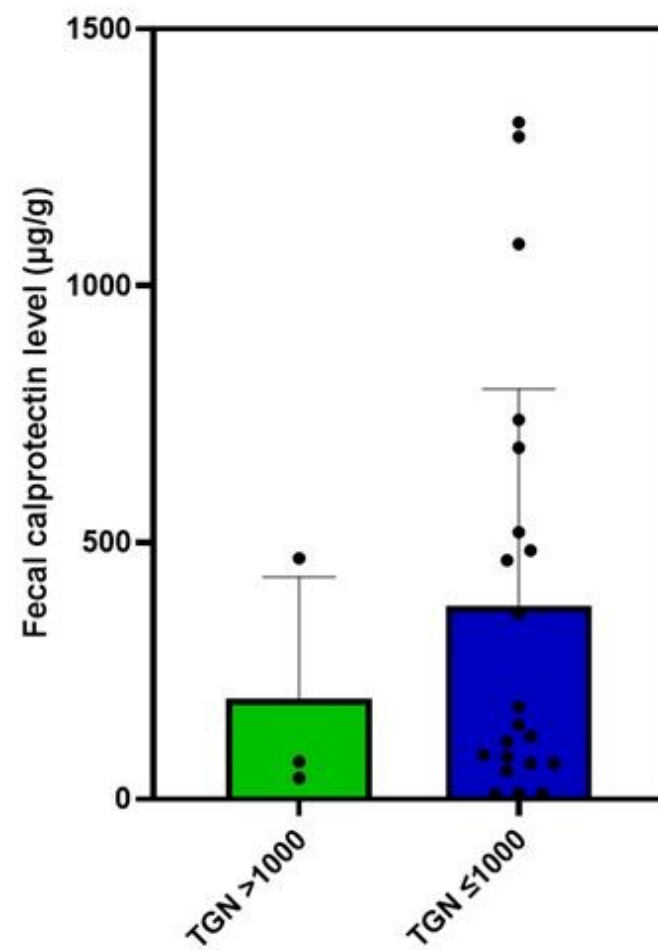

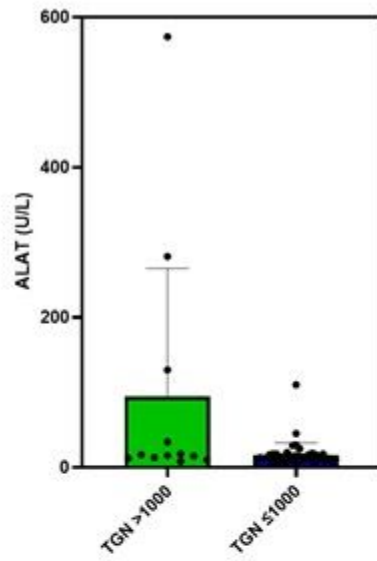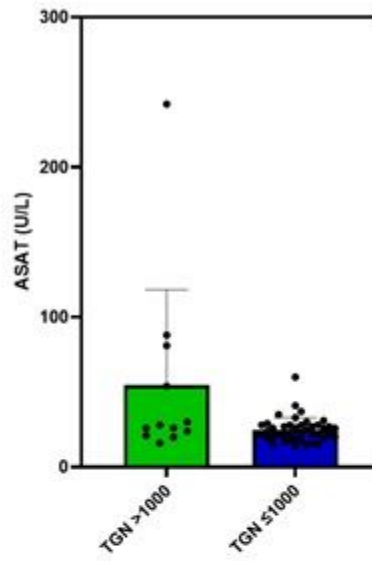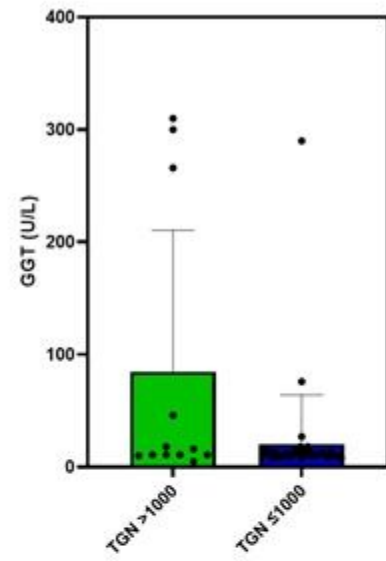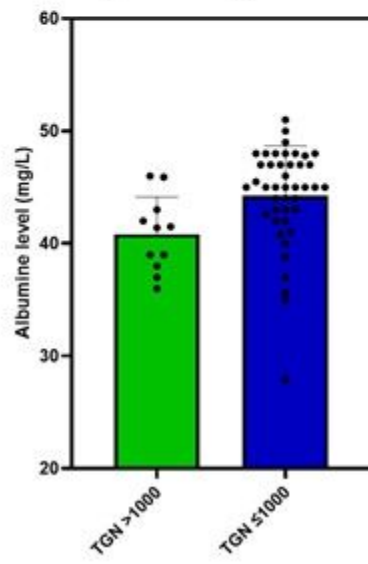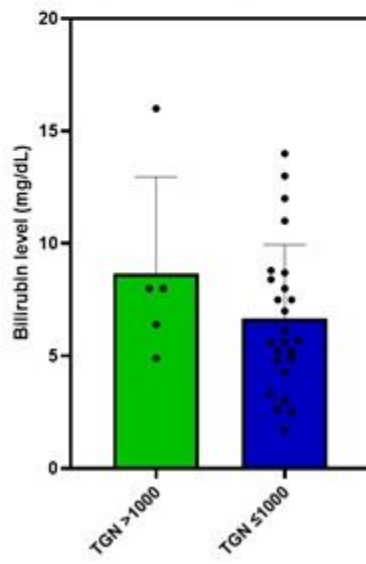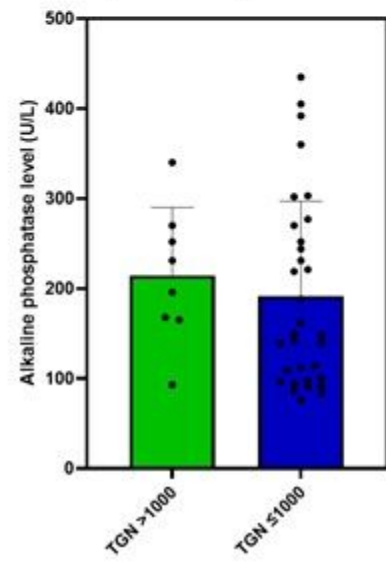

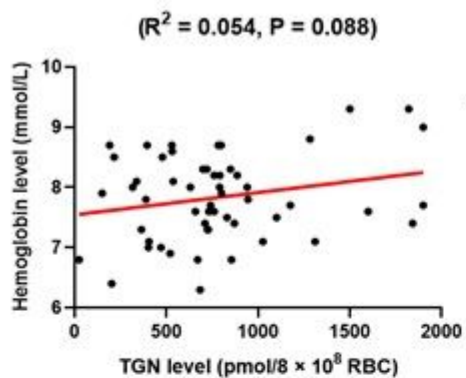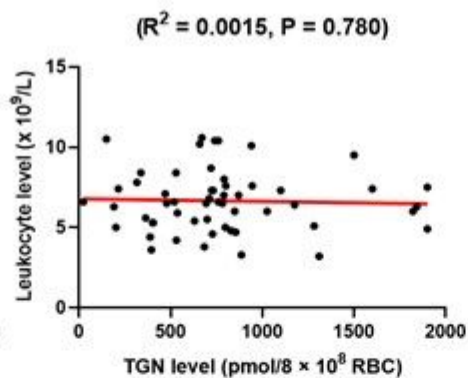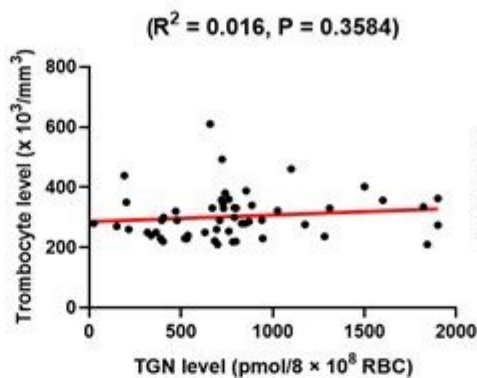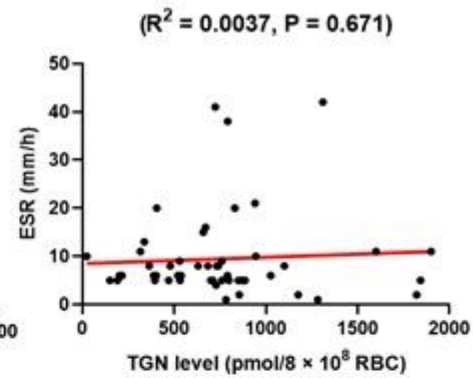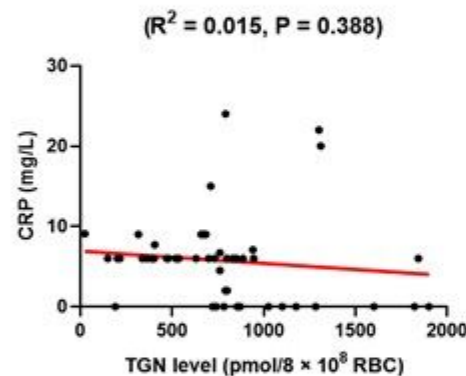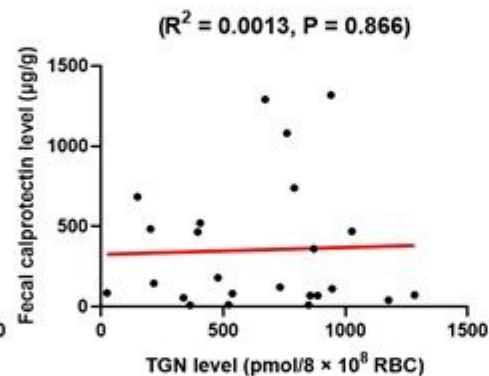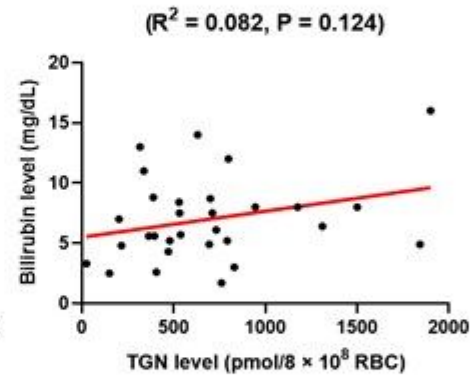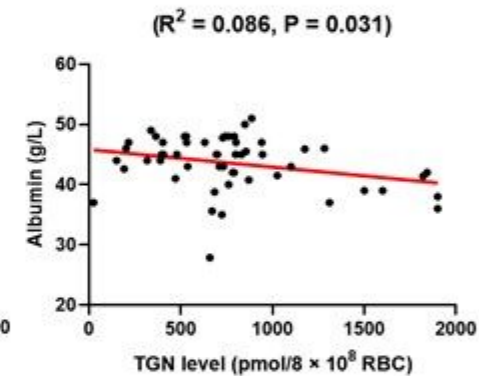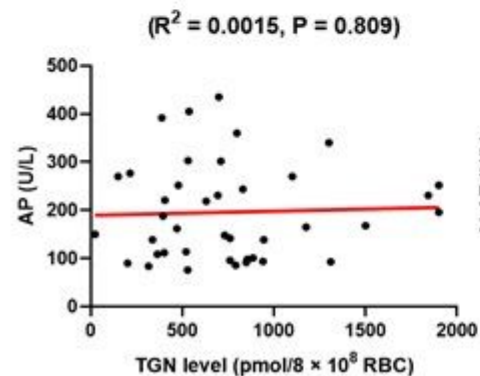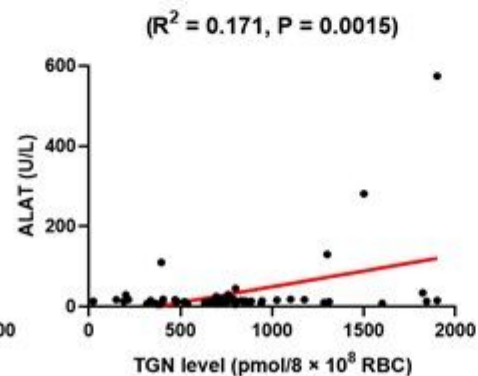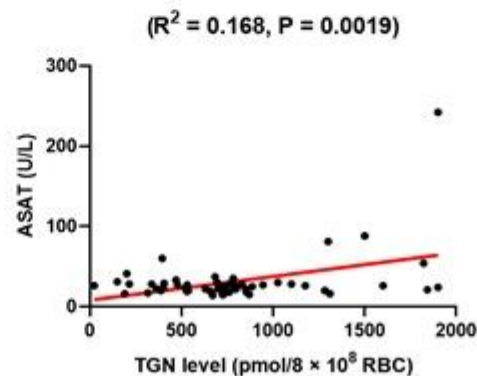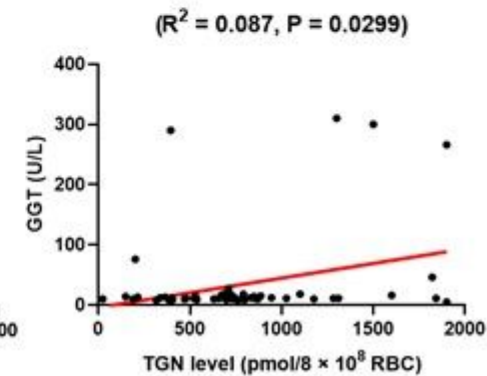

**P = 0.001**

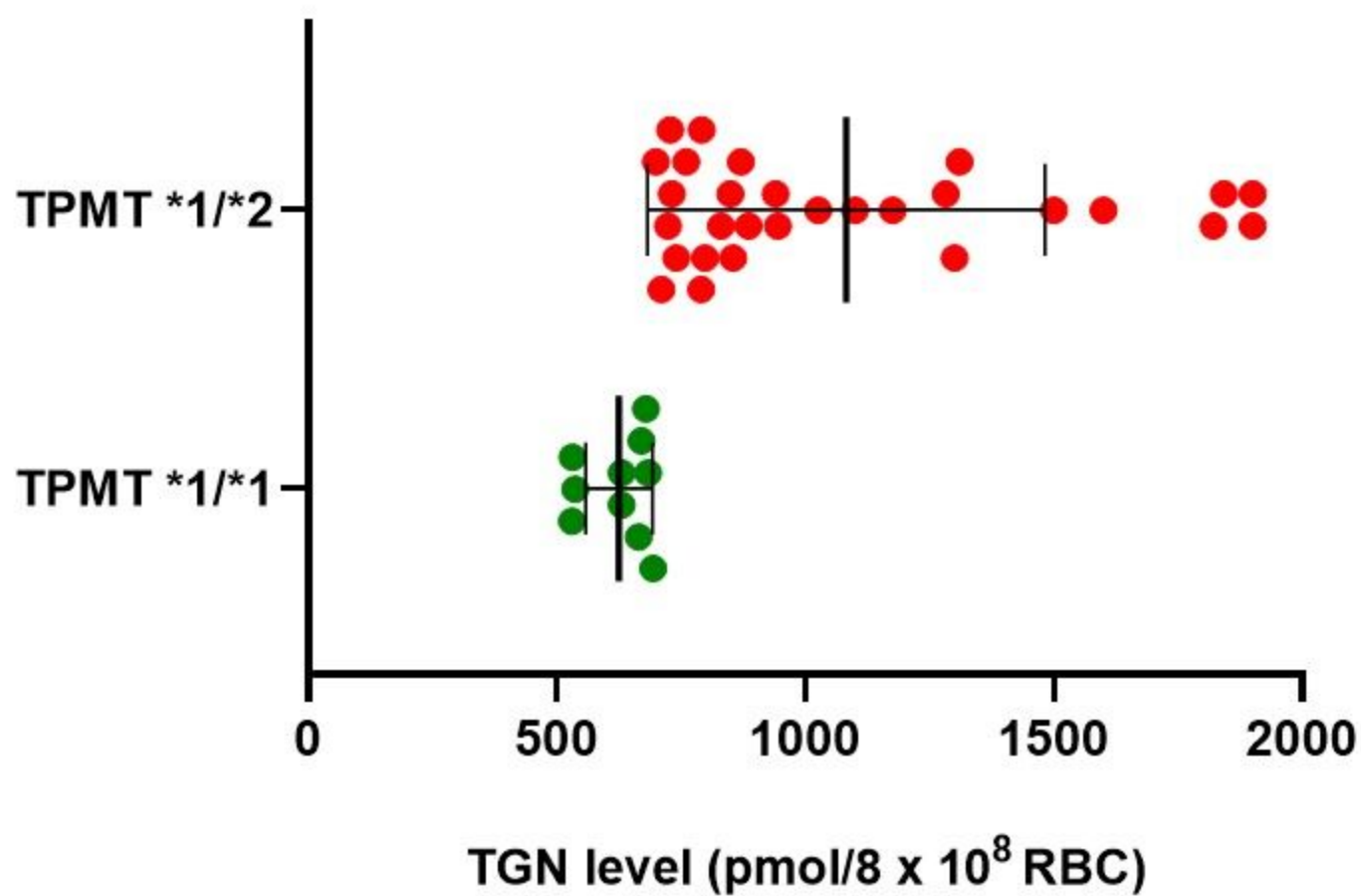

Supplement: Supplementary file 1 [file mpg-75-e111-s001.pdf]
